# Comparison of oral and vaginal metronidazole for treatment of bacterial vaginosis in pregnancy: impact on fastidious bacteria

**DOI:** 10.1186/1471-2334-9-89

**Published:** 2009-06-10

**Authors:** Caroline M Mitchell, Jane E Hitti, Kathy J Agnew, David N Fredricks

**Affiliations:** 1 Department of Obstetrics & Gynecology, University of Washington, Seattle, WA, USA; 2 Fred Hutchinson Cancer Research Center, Seattle, WA, USA

## Abstract

**Background:**

Bacterial vaginosis (BV) is a common condition that is associated with preterm birth and acquisition of complex communities of vaginal bacteria that include several fastidious species. Treatment of BV in pregnancy has mixed effects on the risk of preterm delivery, which some hypothesize is due to variable antibiotic efficacy for the fastidious bacteria. Both oral and intravaginal metronidazole can be used to treat bacterial vaginosis in pregnancy, but little is known about the impact of different routes of antibiotic administration on concentrations of fastidious vaginal bacteria.

**Methods:**

This was a sub-study of a larger randomized trial of oral versus vaginal metronidazole for treatment of BV in pregnancy. Fifty-three women were evaluated, including 30 women who received oral metronidazole and 23 who received intravaginal metronidazole. Bacterial taxon-specific quantitative PCR assays were used to measure concentrations of bacterial vaginosis associated bacterium (BVAB) 1, 2, and 3, *Gardnerella vaginalis, Atopobium *species, *Leptotrichia/Sneathia *species, *Megasphaera *species, and *Lactobacillus crispatus *before and after antibiotic treatment.

**Results:**

Concentrations of *Leptotrichia *and *Sneathia *spp. and the fastidious Clostridia-like bacterium designated BVAB1 decreased significantly with oral (p = .002, p = .02) but not vaginal therapy (p = .141, p = .126). The fastidious bacterium BVAB3 did not significantly decrease with either treatment. Concentrations of *Atopobium *spp., reportedly resistant to metronidazole *in vitro*, dropped significantly with oral (p = .002) and vaginal (p = .001) treatment. There was no significant difference in the magnitude of change in bacterial concentrations between oral and vaginal treatment arms for any of the bacterial species. *Lactobacillus crispatus *concentrations did not change.

**Conclusion:**

Both oral and vaginal metronidazole therapy in pregnant women result in a significant decrease in concentrations of most BV-associated anaerobic bacteria, with the exception that *Leptotrichia, Sneathia *and BVAB1 do not significantly decrease with vaginal metronidazole therapy. These data suggest that the route of antibiotic administration has a minor impact on bacterial eradication in pregnant women with BV.

**Trail Registration:**

This trial is registered with ClinicalTrials.gov, number NCT00153517

## Background

Bacterial vaginosis (BV) is a common cause of vaginal discharge, with a prevalence of 29% in the general population[1]. BV is characterized by a loss of the normal, hydrogen peroxide (H_2_O_2_)-producing vaginal lactobacilli and an increase in the presence of anaerobic bacteria. The sequelae of BV can be serious; pregnant women with BV diagnosed between 8 and 17 weeks gestation have up to a sevenfold increase in the risk of delivery prior to 37 weeks[2].

The efficacy of oral metronidazole for treatment of BV has been reported to range from 87–92% when evaluated 4 weeks after treatment, compared to 61–94% for vaginal metronidazole[3]. However, recurrence rates as high as 70% have been noted one year after treatment[4]. In women with a history of preterm delivery some studies show a decreased risk of preterm delivery after screening for and treatment of BV in early pregnancy[5]. In low risk women, however, no change in the risk of preterm delivery was seen[6].

Recently, bacterial identification using broad range 16S rRNA gene PCR has demonstrated that the vaginal microbiota in subjects with BV is more complex than has been revealed by cultivation methods [7-9]. Not only is BV heterogeneous, with different populations and concentrations of bacteria in different individuals, but previously uncultivated bacteria are highly prevalent in women with BV. Since these bacteria are difficult to culture, their susceptibility to antibiotics is not known at this time. Most treatment studies in pregnancy have used oral metronidazole, though vaginal metronidazole is an acceptable option [10,3] that has shown efficacy against fastidious bacteria[11]. One hypothesis for the lack of effect of metronidazole treatment on rates of preterm delivery is that the route of antibiotic delivery may affect treatment efficacy for some vaginal bacteria. For instance, oral delivery of metronidazole may not eradicate some fastidious BV-associated bacteria if oral treatment results in lower vaginal antibiotic concentrations. To explore this hypothesis, we evaluated vaginal fluid samples from a randomized trial of oral versus vaginal metronidazole to examine the effect of each formulation on quantities of fastidious BV-associated bacteria.

## Methods

**Figure 1:**
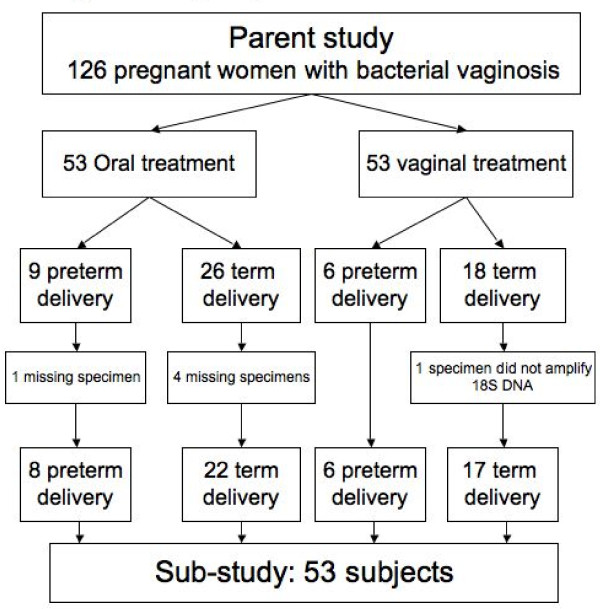
**This paper reports results of a nested case-control sub-study of women who were participants in a randomized controlled trial of oral versus vaginal metronidazole treatment for bacterial vaginosis in the first trimester of pregnancy**. This flow diagram shows how sub-study cases (women with preterm delivery) and controls (women with term delivery) were chosen from parent study groups randomized to oral or vaginal metronidazole treatment, with reasons for subject exclusion.

This study analyzed samples collected during a prospective randomized trial comparing cure of BV with vaginal versus oral metronidazole treatment for asymptomatic bacterial vaginosis in early pregnancy that was conducted between May 2000 and September 2004 in Seattle, Washington. Women were eligible for the parent study if they had a singleton, live, intrauterine pregnancy between 10–20 weeks, were able to provide informed consent and were diagnosed with bacterial vaginosis by Nugent's Gram stain criteria[12] at a screening visit. For this substudy, we enriched our sample set for women likely to experience poor response to treatment by selecting all women who delivered preterm (< 37 weeks). We then selected 3 additional women who delivered at term, matched for race and treatment arm, for each woman who delivered pre-term (Figure 1). The parent study and this substudy were approved by the University of Washington Institutional Review Board (IRB); the parent study was also approved by the Centers for Disease Control and Prevention IRB and was registered with http://www.clinicaltrials.gov, #NCT00153517.

Women were screened at an antenatal clinic visit, then randomized to either oral metronidazole 250 mg three times a day for 7 days with a vaginal placebo, or to 0.5 g of 0.75% vaginal metronidazole twice a day with an oral placebo. Women were assessed at 4 weeks and 8 weeks after treatment, and at delivery. Data on Amsel's clinical criteria for BV [13] were collected at baseline and at all follow-up visits. At each visit four vaginal swabs were collected using standard Dacron swabs: one used for repeat Gram stain, and three others that were placed in 900 uL of phosphate buffered saline or normal saline and frozen at -80°C.

Frozen vaginal swabs from the randomization visit and one post-treatment visit were thawed, mixed by vortex shaker for 1 minute and then removed from the liquid. The liquid was centrifuged for 10 minutes at > 10,000 × g, and the supernatant removed. The remaining pellet underwent DNA extraction with the MoBio UltraClean Soil DNA Isolation Kit (MoBio, Carlsbad, CA). A clean swab was run through the DNA extraction process as an extraction control for each set of samples. All extracted DNA was tested in a quantitative PCR using primers targeting the human 18S rRNA gene to validate that successful DNA extraction occurred. An internal amplification control PCR using exogenous DNA from a jellyfish gene was used to test for presence of PCR inhibitors[14].

Vaginal fluid samples were then subjected to eight separate taxon-directed 16S rRNA gene quantitative PCR assays for the detection and quantification of individual bacteria which have been described elsewhere [11]. One assay detects two bacterial species (*Leptotrichia *and *Sneathia*) that are closely related. Each assay has previously been validated and proven to be sensitive (to a level of 1–10 DNA copies/reaction) and specific (does not detect other bacteria at a concentration of 10^6^copies/rxn). The assays use a TaqMan format, and are run on an ABI 7500 Thermocycler (Applied Biosystems, Foster City, CA). Negative assays were assigned a value at the lower limit of detection for that assay, and were included in all analyses, including the calculation of mean bacterial concentrations.

Statistical analysis was performed using SPSS version 11. Demographic data was compared between groups using the Chi-square test for categorical variables and the Mann-Whitney U test for continuous variables. The change in detection of bacteria (i.e. bacteria present pre-treatment but not post-treatment) was evaluated using the Chi-squared test. Bacterial concentrations were log transformed and comparison of the mean difference in bacterial concentrations before and after treatment within each treatment group was performed using a paired T-test. Comparison of mean log-transformed concentrations pre and post-treatment between the two treatment groups, as well as the change in concentration after treatment was performed using an independent samples T-test. We estimated that we would be able to detect a .95 log difference in the mean change in concentrations of bacteria between women randomized to oral verus vaginal treatment.

## Results

In the parent study, 15 women delivered preterm (< 37 weeks gestation) and 14 had adequate samples for inclusion in this sub-analysis. Forty-four women who delivered at term, matched for treatment assignment and race were selected. Of those, 4 did not have a complete set of pre and post-treatment samples for analysis, and were excluded. One additional subject who delivered at term was excluded because no human rRNA gene DNA was amplified from the post-treatment sample, suggesting that DNA extraction was inadequate or the swab did not contact a human surface. This left a total of 53 women in the final population, 30 of whom had been randomized to oral treatment (8 pre-term and 22 term deliveries) and 23 to vaginal treatment (6 pre-term and 17 term deliveries) (Figure 1). Three women did not have samples available from the 4-week follow-up visit, and so samples collected 8 weeks after treatment were analyzed. There were more African American participants in the oral treatment group, and more Hispanic participants in the vaginal treatment group (Table 1). Women in the oral treatment group were of higher gravidity, but the groups were otherwise similar in terms of gestational age at enrollment and known risk factors for BV, including smoking and douching.

**Table 1:** Demographic data for women randomized to oral or vaginal metronidazole.

	Oral treatment (n = 30)	Vaginal treatment (n = 23)	P value^a^
Age (mean years)	23	20	.17
Race^b^			< .01
*White*	7 (20%)	7 (30%)	
*African American*	13 (43%)	4 (17%)	
*Asian/PI*	10 (33%)	4 (17%)	
*Hispanic*	0	7 (30%)	
Marital status^c^			.60
*Single*	12 (40%)	8 (35%)	
*Married/living with*	17 (57%)	15 (65%)	
Occupation^c^			.65
*Student*	7 (23%)	8 (35%)	
*Employed*	13 (43%)	8 (35%)	
*Unemployed*	10 (33%)	7 (30%)	
Education (highest completed)^c^			.20
*Primary*	9 (30%)	10 (43%)	
*Secondary*	12 (40%)	4 (17%)	
*Tertiary*	9 (30%)	9 (39%)	
History of douching	6 (20%)	2 (9%)	.44
Smoker	11 (37%)	11 (48%)	.41
Gravidity (median)	2	1	.02
Gestational age at enrollment (median in weeks)	15	16	.51
Gestational age at delivery (median in weeks)	39	40	.78
Birthweight (mean in grams)	3160.5	3203	.94
Clinical cure after treatment (ie. 0 Amsel's criteria)	17 (57%)	14 (61%)	.73
Microbiologic cure after treatment (ie. Nugent score ≤ 3)	16 (53%)	12 (52%)	.58

^a^Categorical variables were analyzed using Chi square or Fisher's exact test. Continuous variables were analyzed using the Mann-Whitney U test.

^b^Percentages may not add up to 100% because of rounding, or missing data points

^c^Missing data for some participants

At visit 1, the most prevalent bacteria detected included *Gardnerella vaginalis *(98%), *Atopobium spp*. (73%) *Megasphaera *(70%), *Leptotrichia/Sneathia *(55%) and BVAB2 (51%). Other bacteria were less common: BVAB1 (30%) and BVAB3 (17%). Of women with bacteria detected, there were no differences in rates of bacterial persistence (defined as presence of the a bacterium in both the pre and post-treatment samples) between the two treatment groups (Table 2). *Lactobacillus crispatus*, a marker of vaginal health, was rarely present either before or after treatment (6/53 vs 9/53).

**Table 2:** Persistent detection of individual bacteria by species-specific qPCR after treatment with oral or vaginal metronidazole (Chi-square test)

Bacteria	Oral n/N (%)	Vaginal n/N (%)	P value
*Gardnerella vaginalis*	24/30 (80%)	16/22 (73%)	.43
*Megasphaera *spp.	12/22 (55%)	5/15 (33%)	.38
*Leptotrichia/Sneathia*	6/18 (33%)	5/11 (45%)	.76
*Atopobium vaginae*	11/21 (52%)	9/17 (53%)	.85
BVAB1	0/11 (0%)	1/5 (20%)	.29
BVAB2	3/18 (17%)	1/9 (11%)	.30
BVAB3	1/6 (17%)	1/3 (33%)	.66

n = number of women with bacteria detected at visit 2

N = number of women with bacteria detected at visit 1

The decreases in concentrations of BVAB1 and *Leptotrichia/Sneathia *were statistically significant in those subjects receiving oral therapy, but not in the vaginal treatment subgroup (Table 3). Neither oral nor vaginal treatment caused a significant decrease in concentrations of BVAB3. For the remaining bacteria, treatment was highly effective at decreasing vaginal bacterial concentrations (Table 3). Concentrations of *Atopobium spp *decreased by a mean of 1.92 log (p < .01) in the oral group and 2.12 log (p < .01) in the vaginal group, which was not statistically significantly different. When pre and post-treatment bacterial concentrations were compared between treatment groups, no significant differences were found. *Lactobacillus crispatus *concentrations were not affected by treatment.

**Table 3:** Comparison of the pre- and post-treatment mean log_10 _transformed bacterial concentrations and mean log10 – transformed change in concentration within the groups of women treated with oral and vaginal metronidazole.

Bacteria		Oral (n = 30)	Vaginal (n = 23)
*L. crispatus*	Pre	1.409 ± 1.120^a^	1.495 ± 1.354
	Post	1.798 ± 2.056	2.338 ± 2.726
	Change	.375 (p = .43^b^)	.843 (p = .17)

*G. vaginalis*	Pre	6.939 ± 1.577	6.421 ± 1.962
	Post	5.085 ± 2.449	4.372 ± 2.720
	Change	-1.853 (p < .01)	-2.049 (p < .01)

*Megasphaera *spp.	Pre	4.883 ± 2.551	4.587 ± 2.812
	Post	3.250 ± 2.834	1.970 ± 1.994
	Change	-1.633 (p < .01)	-2.617 (p < .01)

*Leptotrichia/Sneathia*	Pre	3.588 ± 2.203	3.211 ± 2.456
	Post	2.105+2.124	2.331+2.339
	Change	-1.484 (p < .01)	-.880 (p = .14)

*Atopobium*	Pre	4.869 ± 2.567	4.715 ± 2.558
	Post	3.000 ± 2.628	2.598 ± 2.270
	Change	-1.921 (p < .01)	-2.117 (p < .01)

BVAB1	Pre	2.714 ± 2.699	1.909 ± 2.100
	Post	1.337 ± 1.340	1.355 ± 1.321
	Change	-1.378 (p = .026)	-.555 (.127)

BVAB2	Pre	3.343 ± 2.050	2.761 ± 2.294
	Post	1.601 ± 1.603	1.207 ± .992
	Change	-1.742 (p < .01)	-1.554 (p < .01)

BVAB3	Pre	1.773+1.589	1.532 ± 1.447
	Post	1.255 ± .976	1.144 ± .690
	Change	-1.459 (p = .12)	-.765 (p = .10)

^a^All pre- and post-treatment values were compared between groups and were not significantly different.

^b^All p-values are for paired T-test comparing pre-treatment concentration of bacterium to post-treatment concentration of bacterium within the individual treatment group.

## Discussion

Overall this study shows that oral and vaginal metronidazole treatment of bacterial vaginosis in early pregnancy produces comparable changes in most BV-associated bacteria, even fastidious species. A few species, such as *Leptotrichia/Sneathia *and the bacterium BVAB1, show greater response to oral than vaginal treatment. Concentrations of the novel, fastidious bacterium BVAB3 did not decrease significantly in either treatment arm, but this may be due to the small numbers of women with detectable BVAB3 at the first visit: only 6 women in the oral treatment arm and 3 in the vaginal treatment arm.

Subjects with BV have complex communities of vaginal bacteria. Some bacterial species may be directly killed by metronidazole. Other bacterial species may not be susceptible to metronidazole but decrease in concentration because they are metabolically dependent on other species that are susceptible (indirect effects). These data demonstrate that oral metronidazole therapy results in decreased vaginal concentrations of all of these BVABs, though the change in BVAB3 concentration was not statistically significant. None of the fastidious BVAB increased in concentration to suggest that they are resistant to the direct or indirect effects of metronidazole. *Atopobium spp *also showed a significant decrease in bacterial concentrations after treatment despite the observation that many *Atopobium *species are resistant to metronidazole *in vitro*, suggesting an indirect effect [15]. *Lactobacillus crispatus *concentrations did not rise significantly at 4 weeks after treatment.

One limitation of this study is that the post-treatment sample was collected between 4–8 weeks after therapy and may not reflect immediate (and possibly more dramatic) treatment response, though we would expect this to have biased our study not to find a significant change in concentrations. A second limitation is that given the variation in the composition of bacterial populations between women we did not have adequate power to adequately assess the effect of treatment on BVAB3. Our study quantified 8 individual bacterial species. Characterization of total bacterial load and concentrations of additional species would likely provide additional insight into how different communities of vaginal bacteria change in response to antibiotic therapy for BV.

There are many questions that remain unanswered, including whether the intravaginal concentrations of these bacteria reflect their prevalence in the upper genital tract as well, and whether different routes of antibiotic administration alter risk of preterm birth by affecting bacteria in different compartments. With a larger study population we would enhance our ability to determine if a difference in response to treatment for any single bacterium is associated with preterm birth.

Several interesting hypotheses can be generated from these data. First, the lack of significant increase in *Lactobacillus crispatus *concentrations 4 weeks after treatment for BV suggests that the return of normal flora is slow, and that women may be vulnerable to relapse due to the low levels of protective lactobacilli. Second, the significant decrease in *Atopobium *concentrations despite this organism's suspected resistance to metronidazole may suggest that this bacterium and others require the presence of other bacteria in order to thrive in the vaginal environment. Third, although *Leptotrichia*, *Sneathia *and BVAB1 species did not experience the same significant decrease in concentration with vaginal as compared to oral treatment, there was no difference in cure rates, nor in persistence of the bacteria after treatment between the groups. This suggests that the community of anaerobes may be more important than the specific members and that targeting keystone members of the microbial community may have indirect effects on non-target or "antibiotic resistant" bacteria such as *Atopobium vaginae*.

## Conclusion

Both oral and vaginal metronidazole therapy in pregnant women result in a significant decrease in concentrations of most BV-associated anaerobic bacteria, with the exception that *Leptotrichia, Sneathia *and BVAB1 do not significantly decrease with vaginal metronidazole therapy. These data suggest that the route of antibiotic administration has a minor impact on bacterial eradication in pregnant women with BV.

## Abbreviations

BV: bacterial vaginosis; BVAB: Bacterial Vaginosis Associated Bacterium; PCR: polymerase chain reaction

## Competing interests

The authors declare that they have no competing interests.

## Authors' contributions

CM performed the laboratory assays, the primary analysis, and wrote the bulk of the paper. JH was the principal investigator for the parent study, reviewed and approved the analyses and contributed to the writing and revision of the paper. KA performed all of the Gram stains and contributed to writing the Methods section of the paper. DF supervised the laboratory work, reviewed and approved the analyses and contributed to the writing and revision of the paper. All authors read and approved the final version of the manuscript.

## Pre-publication history

The pre-publication history for this paper can be accessed here:

http://www.biomedcentral.com/1471-2334/9/89/prepub
